# Endolymphatic Sac Surgery in Refractory Ménière’s Disease: Exploratory Associations and Postoperative Clinical Outcomes in a Bicentric Cohort

**DOI:** 10.3390/audiolres16010015

**Published:** 2026-01-20

**Authors:** Eleonore Lebelle, Maria-Pia Tuset, Ralph Haddad, Dario Ebode, Daniel Levy, Laetitia Ros, Quentin Mat, Mary Daval, Justin Michel, Laure De Charnace, Stéphane Gargula

**Affiliations:** 1 Department of Otorhinolaryngology, La Conception University Hospital, Aix Marseille University, Assistance Publique Hôpitaux de Marseille (AP-HM), 13005 Marseille, France; 2 Department of Otorhinolaryngology, Head and Neck Surgery, Hospital Fondation Adolphe de Rothschild, 25 Rue Manin, 75019 Paris, France; 3 Department of Otorhinolaryngology, La Conception University Hospital, Aix Marseille University, AP-HM, Centre National de la Recherche Scientifique (CNRS), Institut Universitaire des Systèmes Thermiques Industriels (IUSTI), 13005 Marseille, France; 4 Department of Otorhinolaryngology, Centre Hospitalo-Universitaire Charleroi, 6042 Charleroi, Belgium

**Keywords:** Ménière’s disease, cohort studies, endolymphatic sac, endolymphatic hydrops, hearing, prognosis, vertigo

## Abstract

**Background/Objectives**: Endolymphatic sac surgery (ELSS) is a non-destructive surgical option for medically refractory Ménière’s disease (MD), yet factors influencing surgical outcomes remain poorly understood. This exploratory study aimed to describe clinical outcomes following ELSS and identify potential associations between preoperative characteristics and surgical success. **Methods**: This retrospective, bicentric cohort study included 45 patients with definite MD who underwent ELSS (predominantly endolymphatic duct blockage) between 2019 and 2024. Vertigo control was assessed using AAO-HNS criteria. Hearing outcomes were evaluated through pure-tone and speech audiometry. Univariate analyses explored associations between demographic, clinical, imaging, and surgical variables and treatment outcomes. **Results**: Surgical success (Class A/B vertigo control) was achieved in 66.7% of patients (95% CI: 51.0–80.0%). In a post hoc exploratory analysis, longer disease duration (>5 years) showed an association with better outcomes (87.5% vs. 55.2%, *p* = 0.029), though this threshold was not prespecified and requires validation. Hearing was preserved in 77.5% of patients at 45-day follow-up but declined progressively to 50% at 2 years. Seven patients developed postoperative Tumarkin attacks, with five requiring non-conservative interventions. ELSS demonstrated low morbidity, with one labyrinthitis as the only significant complication. **Conclusions**: ELSS was associated with vertigo control in two-thirds of patients with refractory MD, with a favorable safety profile. Longer disease duration before surgery may be associated with improved outcomes, though this exploratory finding requires confirmation in prospective studies. The progressive hearing decline may reflect both natural disease progression and potential surgical effects. Further research with larger cohorts is needed to establish robust predictive criteria for patient selection.

## 1. Introduction

Meniere’s disease, described by Prosper Ménière in 1861, is defined by episodes of vertigo lasting from 20 min to 12 h associated with unilateral sensorineural hearing loss (allowing identification of the affected side), as well as fluctuating symptoms such as tinnitus or aural fullness [1]. First-line management is medical and includes the use of diuretics, betahistine, and symptomatic treatment for nausea and vomiting induced by vertigo, which achieve complete or partial symptom control in around 80% of cases [2]. Alternative options may be offered to patients who do not respond to this initial medical treatment, such as intratympanic corticosteroid injections [3]. In case of failure of medical treatment, patients may be considered refractory, and offered more radical treatments such as chemical labyrinthectomy with gentamicin injections, or surgical procedures [4].

Although the pathophysiological mechanisms are not yet fully understood, endolymphatic hydrops (a pathological distension of the endolymphatic compartment of the inner ear) has been strongly associated with Meniere’s disease [5]. In a meta-analysis of 541 temporal bones, Foster et al. found histological evidence of endolymphatic hydrops in 97% of patients with Meniere’s disease [6]. Saccular hydrops is most specifically associated with Meniere’s disease, both histologically and on delayed post-contrast MRI sequences [7,8,9]. Consequently, some surgical techniques have been designed to target the endolymphatic fluid circulation system: the endolymphatic sac and duct [10,11,12]. Although this technique carries a relatively high risk of being ineffective compared to ablative options (vestibular neurectomy, chemical or surgical labyrinthectomy), endolymphatic sac surgery has the advantage of being non-destructive, thereby preserving both vestibular and auditory function [13,14,15,16]. This preservation of function makes endolymphatic sac surgery an attractive option in the stepwise management of refractory Menière’s disease, particularly for patients who wish to avoid definitive vestibular ablation or who retain serviceable hearing. Several surgical techniques have been described in the literature to target the endolymphatic system, including endolymphatic sac decompression (ESD), shunt (ESS), or endolymphatic duct blockage (EDB) [17].

Despite renewed interest in endolymphatic sac surgery techniques, particularly endolymphatic duct blockage, and advances in imaging-guided patient selection, factors that may predict surgical success remain poorly defined [8,18,19]. Several recent studies have reported vertigo control rates ranging from 63% to 96%, but the variability in outcomes and the lack of consistent predictive criteria highlight the need for further investigation [20,21,22,23,24]. Understanding which patient characteristics or disease features are associated with better outcomes could improve patient counseling and surgical decision-making.

The primary objective of this exploratory study was to investigate potential associations between preoperative demographic, clinical, and imaging characteristics and the outcomes of endolymphatic sac surgery in patients with intractable Menière’s disease. The secondary objective was to describe the postoperative clinical evolution of patients in our cohort in terms of vertigo control and hearing preservation.

## 2. Materials and Methods

This study is a retrospective, observational, bicentric cohort study. The medical records of all patients who underwent endolymphatic sac surgery (regardless of the specific technique used) between 2019 and 2024 in two tertiary-referral hospitals were reviewed. Ethical approval was obtained from the UMR-T24 IFSSTAR ethics committee, under reference number PADS25-186. In accordance with national regulations, no specific written informed consent was required for this study using anonymized data collected during routine clinical care, and no additional procedures were performed.

### 2.1. Patients

All patients met the criteria for definite Meniere’s disease according to the 2015 AAO-HNS classification [1]. They were all refractory to medical treatment (associations of betahistine, diuretics, hyperosmolar therapy, acetylleucine and/or meclozine), and had previously undergone intratympanic corticosteroid injections before being considered for surgical intervention. In both centers, this stepwise management reflects a conservative strategy aiming to preserve inner ear function, with endolymphatic sac surgery positioned as the preferred next step before resorting to ablative or destructive options. Vestibular testing (caloric testing and video head impulse testing) was performed as part of the routine diagnostic assessment in all patients; however, vestibular test results were not used to guide surgical decision-making and were not considered suitable for quantitative analysis in this retrospective cohort of patients with severe and refractory Ménière’s disease. Consequently, vestibular test results were not included in the outcome analyses. All patients underwent preoperative delayed post-contrast MRI, which revealed saccular hydrops in all patients [8,18]. MRI studies were interpreted by neuroradiologists and ENT surgeons at each center. Radiological assessments were performed as part of routine clinical care and were not blinded to clinical presentation. Patients with insufficient follow-up duration (less than 6 months postoperatively at the time of analysis) or those lost to follow-up before 6 months after surgery were excluded from the final analysis. A non-serviceable hearing was not considered a criterion for excluding patients from endolymphatic sac surgery at our centers.

### 2.2. Surgical Techniques

Several surgical techniques were used, depending on the patient’s anatomy and the surgeon’s experience. Endolymphatic sac decompression (ESD) involved exposure of the sac through mastoidectomy and drilling of the subfacial air cells. When the sac was opened and partially shunted, the procedure was referred to as endolymphatic sac shunt (ESS), with or without insertion of silastic material. In some cases, duct blockage (EDB) was performed by clipping or sectioning the endolymphatic duct followed by the insertion of a biological glue–muscle mixture to isolate the sac [19]. The distribution of surgical techniques in our cohort was highly imbalanced, with the majority of patients undergoing EDB. This study should therefore be primarily interpreted as an EDB-dominant case series. All surgical techniques (ESD, ESS, and EDB) were analyzed together to provide a descriptive comparison of outcomes. Given the anticipated imbalanced distribution and limited sample sizes in technique subgroups, any comparisons between techniques were planned as exploratory only, and no formal statistical superiority claims between techniques are made in this manuscript.

### 2.3. Evaluation of Surgical Efficacy

The efficacy of surgery was assessed based on the reduction in the frequency of vertigo attacks. For this purpose, we used the clinical index defined by the AAO-HNS in its guidelines for the diagnosis and evaluation of therapies in Meniere’s disease [25]. This index was calculated using the formula R = (X)/(Y) × 100, where X represents the average number of vertigo attacks per month between the 18th and 24th month postoperatively, and Y represents the average number of vertigo attacks per month during the 6-month preoperative period. For patients with a follow-up duration between 6 and 24 months, X was defined as the average number of vertigo attacks per month during the last 6 months of follow-up.

Patients were classified into six categories of vertigo control according to the value of this ratio, as defined in the AAO-HNS guidelines [26]. Patients who required a second surgical procedure were classified as class F, regardless of their clinical status following the first intervention. No patients in our cohort were classified as class E. For analysis purposes and account for the small number of patients in intermediate classes (C and D), the categories A–B (R = 0–40) and C–D–F (R > 40) were grouped together to define “Success” and “Failure”.

### 2.4. Hearing Assessment

For each patient, preoperative and postoperative pure-tone and speech audiometry results were collected. Preoperative hearing was defined using the worst audiogram available within the 6 months prior to surgery. The pure-tone average (PTA) was calculated as the mean of thresholds at 500, 1000, 2000, and 4000 Hz, in accordance with WHO international guidelines [27]. Speech audiometry was reported as the percentage of correctly repeated words from a standard disyllabic word list presented at 60 dB. For each patient, the stage of hearing impairment (as defined by the WHO) was determined based on the preoperative audiogram [28].

The shape of the pure-tone audiometry curve was also recorded and classified into four types: ascending curve (>20 dB difference between the most impaired low frequency and the least impaired high frequency), descending curve (>20 dB difference between the least impaired low frequency and the most impaired high frequency), flat curve (<20 dB difference between the most and least impaired frequencies) bell-shaped curve (>20 dB difference between the best frequency and both the most impaired low and high frequencies).

Postoperative hearing was assessed at each follow-up visit: day 15, day 45, 6 months, 1 year, and 2 years. Information on the presence of hearing fluctuations, tinnitus, and aural fullness in the preoperative period was also collected when available.

### 2.5. Vertigo Assessment

The frequency of vertigo attacks (reported as number of episodes per month), as well as the presence of Tumarkin attacks, was recorded for each patient based on chart review over the 6 months preceding surgery, and then at each follow-up visit (day 15, day 45, 6 months, 1 year, and 2 years). Because vertigo frequency relied on patient self-reports during scheduled consultations, minor or short-lasting episodes may have been underreported. To limit recall bias, patients were systematically encouraged to record each vertigo episode in a personal agenda or diary provided during consultations, noting the date, duration, and intensity of attacks. These records were reviewed by the clinician at each visit and transcribed into the medical chart. This standardized documentation procedure was applied in both centers before and after surgery.

### 2.6. Ct Morphological Assessment

CT imaging of the temporal bone was routinely performed to assess vestibular aqueduct anatomy. For the purpose of the analysis, it was re-interpreted by one neuroradiologist to measure VA width in the axial plane on the first slice where it appeared clearly separated from the dura [29].

### 2.7. Statistical Analysis

This study was designed as an exploratory analysis to investigate potential associations between preoperative variables and surgical outcomes. All statistical analyses were performed using MedCalc^®^ software, version 23.2.1 (MedCalc Software). Given the exploratory nature of this study and the limited sample size (*n* = 45), we conducted univariate analyses only, without correction for multiple comparisons. No primary outcome predictor was prespecified a priori. Therefore, all findings should be interpreted as hypothesis-generating and requiring validation in independent cohorts.

Comparisons of qualitative variables were conducted using the Chi-squared test or Fisher’s exact test when expected cell counts were less than 5. Comparisons of quantitative variables were performed using the *t*-test for normally distributed data or the Mann–Whitney U test for non-normally distributed data. Normality was assessed using the Shapiro–Wilk test. For all statistical tests, *p*-values < 0.05 were considered statistically significant for exploratory purposes. Where appropriate, 95% confidence intervals and effect sizes are reported alongside *p*-values to aid clinical interpretation.

The dichotomization of disease duration at 5 years was performed post hoc based on the approximate median disease duration in our cohort, which ensured balanced subgroup sizes for comparison. This threshold was not prespecified and should not be interpreted as a validated clinical cutoff.

## 3. Results

Fifty-two patients underwent endolymphatic sac surgery during the inclusion period at the two participating centers. After applying exclusion criteria, 45 cases were included, as 7 patients had undergone surgery less than 6 months before the time of analysis. No additional patients were excluded for loss to follow-up or other reasons. The population characteristics are summarized in Table 1. Thirty-four (75%) patients underwent EDB, 10/45 (22.2%) underwent ESS, and one patient underwent ESD. The mean follow-up duration was 20.1 months (range: 6–63.3 months; median: 16.6 months).

Surgical success was observed in 30 patients (22 patients classified as Class A and 8 patients as Class B), representing 66.7% of the cohort. Failure was observed in 15 patients (2 patients classified as Class C, 2 patients as Class D, and 11 patients as Class F), representing 33.3% of the cohort.

### 3.1. Exploratory Analysis of Factors Associated with Surgical Success and Failure

The following analyses are exploratory and hypothesis-generating. No corrections for multiple comparisons were applied, and no primary predictor was prespecified. All findings should be interpreted with caution and require validation in independent cohorts.

#### 3.1.1. Demographic and Clinical Factors

No significant differences were found for the demographic factors studied: sex, side of the operated ear, treatment center, or patient age. The surgical technique did not significantly influence surgical success, as 13 of 34 (38.2%) patients in the EDB group were classified as “failures,” compared with 2 of 11 (18.1%) in the ESS/ESD group (*p* = 0.43; Table 1). Given the small subgroup sizes (ESS: *n* = 10; ESD: *n* = 1), this comparison should be interpreted as descriptive only.

#### 3.1.2. Disease Duration

The mean disease duration did not differ significantly between the two groups (Table 1). However, disease duration was highly heterogeneous among patients, ranging from 1 to 37 years. We therefore conducted a post hoc exploratory analysis to assess whether longer disease evolution could influence surgical outcomes. For this purpose, patients were divided into two subgroups according to whether disease duration was greater or less than five years before surgery (Table 2). The five-year threshold corresponded approximately to the median disease duration in our cohort, ensuring balanced subgroup sizes.

Patients with a disease duration shorter than 5 years had a surgical success rate of 55.2% (16/29), whereas those with symptoms lasting longer than 5 years achieved 87.5% success (14/16) (*p* = 0.029; OR = 5.69; 95% CI: 1.15–28.1). In our series, surgery performed within the first 5 years of symptom onset was associated with a higher proportion of failures compared with surgery performed later in the disease course.

#### 3.1.3. Vertigo Characteristics

No significant difference was found regarding the mean preoperative number of vertigo attacks per month (Table 1). An additional analysis was performed comparing patients with less than one attack per week, 1 to 2 attacks per week, or more than 10 attacks per month (Table 3).

This analysis did not reveal any significant difference: the frequency of vertigo attacks does not appear to be a determining factor in surgical success. The presence of associated migraine symptoms did not alter the prognosis of the procedure, although the sample size was relatively small (11 patients). The presence of Tumarkin attacks preoperatively was not a predictive factor of surgical success or failure. No significant difference was observed between patients who experienced Tumarkin attacks before surgery, with 7 out of 9 patients (77.8%) in the success group versus 2 out of 9 (22.2%) in the failure group (*p* = 0.43; OR = 3.50; 95% CI: 0.59–20.8; Table 1).

#### 3.1.4. Preoperative Hearing

Regarding hearing outcomes, there was no significant correlation between surgical success and pure-tone average, speech audiometry, or audiometric stage (Table 1).

As Meniere’s disease is typically characterized by fluctuating hearing loss and a low-frequency hearing impairment, we examined the association of these features with surgical outcome. Neither was found to be significant. Ascending audiometric curves, indicating predominant low-frequency hearing loss, were not more frequently associated with surgical success than other curve shapes (Table 4). Similarly, the presence of hearing fluctuations did not appear to be a predictive factor for surgical success or failure (Table 1).

#### 3.1.5. CT Morphology of the Vestibular Aqueduct

CT measurements revealed no morphological differences in the vestibular aqueduct between the surgical success and failure groups (Table 1).

### 3.2. Functional Outcomes

#### 3.2.1. Early Postoperative Vertigo

In our cohort, 24 patients experienced vertigo or instability during the first month after surgery. Of those, 11 exhibited improvement during this period. Conversely, among the patients who did not experience any vertigo in the first postoperative month, only one had an unfavorable outcome. The absence of vertigo in the immediate postoperative period showed a strong post hoc association with subsequent surgical success (OR = 15.0; 95% CI: 1.75–128; *p* = 0.002), whereas the presence of early instability did not reliably predict subsequent evolution (Table 5).

#### 3.2.2. Vertigo During Postoperative Follow-Up

**Figure 1:**
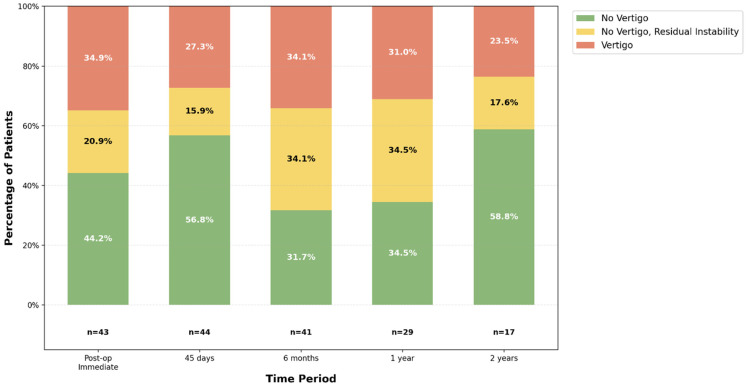
Distribution of Vertigo Symptoms Across Post-Operative Time Periods.

Figure 1 illustrates the progression of vertigo symptoms experienced by patients from the postoperative period up to two years after surgery. The proportion of patients reporting instability or vertigo remained relatively stable from the postoperative period through one year. At two years, the proportion of patients free from vertigo increased. This includes patients whose vestibular function stabilized, but the “failure” group also artificially decreased due to patients classified as Class F who received adjunctive treatments to control their disease.

We also noted that 7 patients in our cohort developed Tumarkin attacks postoperatively. In two of these cases, the attacks were temporary and resolved without the need for additional intervention. For the remaining five patients, these attacks prompted the indication for non-conservative treatment (vestibular neurectomy or intratympanic gentamicin).

#### 3.2.3. Pure-Tone Audiometry Postoperatively

Table 6 presents changes in pure-tone audiometry relative to preoperative baseline values. Missing data increased over time due to loss to follow-up and patients receiving additional interventions.

At postoperative day 15, audiometry was available for 42 patients (93.3%); at day 45, for 40 patients (88.9%); at 6 months, for 37 patients (82.2%); at 1 year, for 30 patients (66.7%); and at 2 years, for 16 patients (35.6%). No imputation method was applied for missing data.

**Figure 2:**
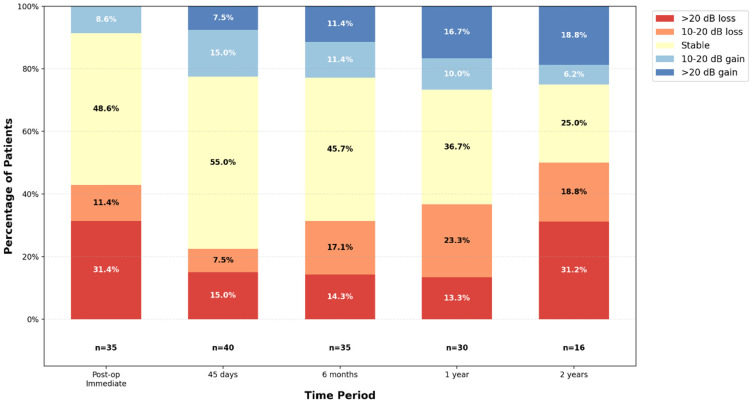
Distribution of Audiometric Changes Across Post-Operative Time Periods.

Figure 2 illustrates the progression of patients’ pure-tone audiometry relative to their preoperative hearing levels. In the immediate postoperative period, a transient worsening of hearing was observed in 42.9% of patients (defined as a loss of over 10 dB compared to the reference preoperative audiogram), with an average loss of 13.9 dB (Table 6). This loss proved to be temporary: at the 45-day postoperative check-up, 77.5% of patients had stable or improved hearing, with an average loss of only 1.6+/−16.5 dB. However, a gradual deterioration was noted up to two years postoperatively: 50% of patients experienced a hearing loss of more than 10 dB, with an average loss of 6.1+/−27 dB.

#### 3.2.4. Speech Audiometry Postoperatively

At the 45-day postoperative assessment, 70.9% of patients had stable or improved speech audiometry compared to their preoperative evaluation, with an average gain of 2.1%. However, a subsequent decline was observed over time. Two years after surgery, speech audiometry had deteriorated in 50% of patients compared to their preoperative baseline, with a mean loss of 18.6%.

#### 3.2.5. Need for Additional Surgical or Non-Conservative Treatment

Eleven patients in our study required supplementary treatments to control their disease after the initial surgery. Three of them underwent revision surgery involving ipsilateral EDB (two early revisions within 3 months postoperatively and one late revision at 1 year). Intratympanic gentamicin injections were administered to two patients, vestibular neurectomy was performed in eight patients, and surgical labyrinthectomy was performed in one patient. Among the three patients who underwent EDB revision surgery, the intervention controlled the disease in one, while the other two required additional non-conservative treatments. One of them subsequently received intratympanic gentamicin injections and later underwent vestibular neurectomy. The interval between initial surgery and the initiation of non-conservative treatment ranged from 4 to 29 months, with a mean of 13.7+/−8.9 months.

#### 3.2.6. Postoperative Complications

In our cohort, three patients experienced immediate postoperative complete hearing loss. They all presented with significantly impaired preoperative hearing (stage 3). Two of these patients partially recovered (10–20 dB lower than preoperative audiometry). They had undergone surgery performed by EDB, and a delayed post-contrast MRI suggested labyrinthitis in both patients. The third patient presented with persistent worsening of hearing loss in the operated ear. This patient had undergone ESS surgery, and delayed post-contrast MRI revealed vestibular atelectasis. Functional tests of this patient revealed complete vestibular deficit associated with hearing loss. This patient subsequently had a favorable outcome in terms of vertigo control (Class A), but experienced a 25 dB loss in pure-tone audiometry and a 10% decline in speech audiometry compared to her preoperative baseline. No cerebrospinal fluid leaks nor facial palsy were observed in our cohort.

## 4. Discussion

### 4.1. Exploratory Findings on Potential Prognostic Factors

Although studies on endolymphatic sac surgery are becoming more numerous, particularly since the description of EDB, little data exists to aid in patient selection and preoperative assessment of the prognosis for success. The primary objective of this study was to identify clinical or paraclinical factors potentially influencing the outcome of endolymphatic sac surgery in patients with Meniere’s disease refractory to medical treatment.

In our univariate exploratory analysis, the only preoperative variable showing a statistically significant association with surgical success was disease duration of more than five years. This finding emerged from a post hoc analysis using a threshold corresponding approximately to the median disease duration in our series, which ensured balanced subgroup sizes for comparison. Given the exploratory nature of this finding, this association should be interpreted as hypothesis-generating rather than definitive. The observed association between longer disease duration and better surgical outcomes is intriguing and warrants careful interpretation. Interestingly, the 5-year timeframe roughly coincides with the period after which several longitudinal studies have reported a spontaneous decline in vertigo activity in Ménière’s disease, although the timing and magnitude of this process remain highly variable across cohorts [30,31,32]. Several hypotheses may explain this observation. First, patients operated earlier in the disease course may represent a subgroup with more active or aggressive forms of Ménière’s disease, potentially less likely to stabilize after surgery. Conversely, those with longer evolution may be entering a less active phase in which vertigo control is easier to achieve, regardless of surgical intervention. We cannot exclude that the apparent benefit of longer disease duration partly reflects the natural history of the disorder (including spontaneous “burn-out”) rather than a true prognostic effect of disease duration itself. Selection bias may also play a role, as patients with particularly severe or refractory disease may have been operated earlier. In contrast, a study published in early 2025 by Derieppe et al., which aimed to identify predictors of surgical outcomes in Menière’s disease, did not find any factors associated with surgical success [20,21]. In their study, the interval between the onset of the first symptoms and surgery was not identified as a factor influencing surgical outcomes, contrary to our findings.

The association between absence of early postoperative vertigo and long-term success observed in our study (OR = 15.0; *p* = 0.002) likely reflects early treatment response rather than an independent prognostic factor. Patients who remain asymptomatic in the immediate postoperative period may simply be responding favorably to the intervention from the outset. This finding should not be interpreted as a predictive criterion that could be assessed preoperatively for patient selection. Rather, it may serve as an early indicator of treatment efficacy that could inform discussions about the need for additional interventions in patients with persistent early symptoms.

Regarding surgical technique, our study does not provide adequate data to support conclusions about comparative efficacy between EDB, ESS, and ESD. The highly imbalanced distribution (EDB: 75.6%; ESS: 22.2%; ESD: 2.2%) and small subgroup sizes render any statistical comparisons underpowered and potentially misleading. While we observed numerically higher failure rates in the EDB group compared to ESS/ESD (38.2% vs. 18.2%, *p* = 0.43), this difference was not statistically significant and the confidence intervals were extremely wide (OR = 0.45; 95% CI: 0.08–2.47). Therefore, no claims of equivalence or superiority between techniques can be made based on our data. Our study should be interpreted primarily as an EDB-dominant case series with descriptive information on a small number of ESS procedures. Other preoperative factors investigated, including vertigo attack frequency, presence of Tumarkin attacks, hearing levels, audiometric curve shape, hearing fluctuations, and vestibular aqueduct morphology, did not show statistically significant associations with surgical outcomes in our exploratory analyses. However, the absence of significant findings should not be interpreted as definitive evidence of no effect, given the limited sample size and the exploratory nature of our analyses.

### 4.2. Vertigo Control

The rate of surgical success (Class A/B vertigo control) in our study was 66.7%, combining multiple surgical techniques (ESD, ESS, EDB). This success rate is consistent with current literature [16,20,22]. Derieppe et al. reported a 63% success rate at 2 years in patients who underwent sac ESD alone [20,21]. A 2014 meta-analysis of 36 studies published between 1983 and 2012 found a success rate (defined as Class A/B vertigo control) of 79.3% at 1 year and 81.6% at 2 years for decompression, and 76.4% at 1 year and 75.7% at 2 years for ESS [23]. Saliba et al., in a randomized controlled study, reported a higher success rate of 96.5% for EDB [24]. However, important differences exist between their study population and ours, particularly in terms of preoperative disease severity: Saliba et al.’s patients experienced an average of 8.4 vertigo episodes in the 6 months before surgery (1.4/month), whereas our patients averaged 6.7 episodes per month. This disparity in baseline severity may contribute to the differences in outcomes. Nevertheless, our study did not find a statistically significant difference between patients with fewer than one vertigo attack per week and those with more than 10 attacks per month (Table 3), although the number of patients in these extreme categories was small.

This observation highlights the differing approaches to endolymphatic sac surgery within the therapeutic strategies for Ménière’s disease across medical teams. In our two centers, sac surgery was considered a last-resort option for patients with severe symptoms unresponsive to multiple lines of medical treatment. All patients had been symptomatic for over a year and had undergone medical therapy followed by one or more series of intratympanic corticosteroid injections before being offered surgery. In contrast, Saliba et al. proposed surgery after six vertigo episodes within six months and persistent vertigo despite six months of medical therapy, thus targeting a slightly different patient population.

Comparative data among endolymphatic sac procedures remain limited. To date, the only published randomized trial (Saliba et al. 2015) [24] compared endolymphatic duct blockage (EDB) to traditional sac decompression (ESD) and found significantly better vertigo control with EDB More recently, Jiang et al. conducted observational studies comparing EDB, decompression, and sac shunting (“drainage”) within the same cohort [33]. These studies similarly suggest that EDB and sac shunting outperform decompression alone in terms of vertigo control, with EDB potentially offering superior long-term preservation of hearing and vestibular function. In our series, most patients underwent either EDB or ESS, while only one received ESD. These procedures were therefore analyzed descriptively, with ESS and ESD grouped as classic approaches. Although subgroup sizes did not permit robust statistical comparison, this exploratory analysis contributes new comparative data to a sparsely documented area of the literature.

Another aspect that may contribute to heterogeneity between studies is the choice of outcome measures. The study by Schenck et al. showed complete resolution of seizures in 73% of patients who underwent surgery. However, if the criterion for success was defined as improvement in patients’ quality of life, this figure rose to 88% [22]. In our study, the use of the AAO-HNS criteria, while providing rigorous analysis, does not take into account a proportion of patients who continued to experience vertigo spells, albeit of lesser duration and intensity, and who did not consider themselves to be in need of additional treatment (groups C and D). This underlines the gap that may exist between objective vertigo control and patients’ subjective perception of improvement. Because of the retrospective design, no standardized quality-of-life or patient-reported outcome measures were available in our series. Nevertheless, recent prospective studies, including the second part of Derieppe et al.’s 2025 series, have demonstrated significant postoperative improvement in quality-of-life scores after endolymphatic sac surgery and [21]. These findings suggest that the perceived benefit of surgery may exceed what is captured by purely objective vertigo control criteria and emphasize the importance of integrating validated patient-reported outcomes in future studies.

Finally, the contribution of natural disease course to observed outcomes must be acknowledged. Part of the improvement observed after surgery may reflect the natural evolution of Ménière’s disease rather than the surgical intervention itself. This limitation is inherent to all interventional studies on Ménière’s disease and stems from the unpredictable evolution of the disorder and the possible spontaneous decline in vertigo activity over time, known as the “burn-out” phase [30,31,32]. In our cohort, surgery was offered only to patients with particularly active and refractory disease who had failed multiple lines of medical therapy, which may have partially mitigated, but certainly not eliminated, the possible contribution of natural remission to observed results. The lack of a control group prevents definitive attribution of outcomes to the surgical intervention.

### 4.3. Preservation of Hearing

Preserving hearing is a key goal in the treatment of Menière’s disease. At first glance, the mean pure tone audiometry loss of 6.1 dB at 2 years postoperatively, as shown in Table 6, might suggest that surgery has a minimal impact on hearing. Several meta-analyses support this view. For instance, Szott et al. (2022) reported a mean hearing loss of 9.25 dB in 66 patients who underwent ESS [34]. Similarly, Sood et al. (2014) found a mean loss of 6.0 dB at 2 years in patients who also had ESS [23].

However, Figure 2 reveals important temporal dynamics and inter-patient variability. At 45 days postoperatively, 77.5% of patients had stable or improved hearing; this proportion decreased progressively to 68% at 6 months, 63% at 1 year, and 50% at 2 years. Sood et al. observed similar trends, with 71.4% maintaining stable or improved hearing at 1 year and 69.3% at 2 years These findings highlight substantial variability in postoperative hearing outcomes, ranging from complete deafness in one patient to significant improvement (over 30 dB at 2 years) in three others. Notably, more than half of patients who experienced hearing deterioration had losses of less than 20 dB, which may be considered clinically insignificant. However, 13.3% at 1 year and 31.3% at 2 years experienced losses greater than 20 dB, representing clinically meaningful deterioration.

Speech audiometry showed a progressive decline, with 30% of patients experiencing deterioration at 1 year and 50% at 2 years. The mean losses were 10.2% at 1 year and 18.6% at 2 years. These results are consistent with those of Szott et al., who reported a mean loss of 26.23% [34]. Given the gradual and delayed nature of hearing deterioration, distinguishing the consequences of surgical modification of endolymphatic physiology from the natural progression of Ménière’s disease is challenging [30,35,36]. Similarly, while surgery may have improved hearing in some patients, it is possible that preoperative audiometry used as a comparator was performed during a period of acute deterioration, leading to apparent improvement that actually represents regression to baseline. The absence of a control group and the retrospective design limit our ability to definitively attribute hearing changes to surgical intervention versus disease progression.

Regarding the three patients who experienced postoperative hearing loss, it is notable that the only patient who experienced vestibular atelectasis and had the poorest hearing recovery underwent ESS. It seems plausible that the risk of vestibular atelectasis may be higher with shunting procedures due to endolymph depletion, though our limited number of cases precludes definitive conclusions.

### 4.4. Limitations

This study has several important limitations that affect interpretation of our findings. The retrospective and uncontrolled design is the primary limitation. Although patients were instructed to record vertigo episodes prospectively in a personal diary, the final analysis relied on self-reported data transcribed into medical charts, introducing potential recall bias. Minor or brief attacks may have been underreported, though this bias is likely non-directional since the same documentation method was applied consistently before and after surgery in both centers.

The relatively small cohort size (*n* = 45) is a fundamental limitation, though it remains within the range of most recent series on endolymphatic sac surgery (typically 25–50 patients). The small sample size precluded multivariable analysis and may lead to overinterpretation of univariate findings. The exploratory finding regarding disease duration should be interpreted with particular caution given the post hoc nature of the analysis, arbitrary threshold selection at the median (5 years), absence of prespecification, and lack of correction for multiple comparisons. Type I error (false positive finding) remains a significant concern. Future studies should analyze disease duration as a continuous variable and validate any potential threshold effects using larger, independent cohorts.

The limited number of cases in the ESD (*n* = 1) and ESS (*n* = 10) subgroups did not permit robust statistical comparison between surgical techniques. Our study should be interpreted primarily as an EDB-dominant case series with descriptive information on ESS. Any suggestion of comparative efficacy between techniques is not supported by our data. Follow-up duration presents another limitation. While AAO-HNS recommends a minimum two-year follow-up for evaluating Ménière’s therapies, we accepted a six-month minimum for sample-size considerations [25]. However, the relative stability of vertigo control after the initial 6 months (Figure 1) reduces the likelihood of significant systematic bias from shorter follow-up in some patients. Nevertheless, outcomes in patients with shorter follow-up should be interpreted cautiously as they may not reflect long-term disease control.

Sample attrition over time affects interpretation of hearing outcomes. Audiometry was available for 93.3% of patients at day 15 but only 35.6% at 2 years, due to loss to follow-up and patients receiving additional interventions. No imputation method was applied for missing data. If patients with worse outcomes were more likely to be lost to follow-up or pursue additional treatments, our hearing results may overestimate preservation rates at longer time points.

Although vestibular testing (including caloric testing and video head impulse testing) was performed as part of the routine diagnostic work-up, vestibular test results were not analyzed quantitatively in this study. This limits correlation between postoperative vertigo outcomes and objective vestibular function.

Imaging interpretation was not standardized or blinded. MRI and CT assessments were performed as part of routine clinical care by both neuroradiologists and neurotologists, without independent re-evaluation by observers blinded to clinical outcomes for this retrospective analysis. While all patients demonstrated saccular hydrops on MRI, potentially representing selection bias, this likely reflects institutional practice patterns rather than limiting generalizability, as MRI evidence of hydrops is increasingly considered appropriate for surgical candidate selection. Although post-contrast MRI of endolymphatic hydrops is increasingly used in clinical practice, detailed morphometric characterization of the endolymphatic sac and duct requires highly standardized imaging protocols and remains challenging outside dedicated prospective studies. As emphasized by recent international consensus recommendations, this limits the feasibility of reliable post hoc analyses in retrospective cohorts [37]. Nevertheless, such standardized post-contrast MRI analyses may prove highly valuable in future prospective studies to better characterize ES/ED endotypes and assess their potential role in patient stratification and surgical decision-making.

The inherent heterogeneity of Ménière’s disease regarding clinical presentation and severity represents an additional challenge. Although all patients were diagnosed with unilateral MD, unnoticed contralateral involvement cannot be excluded and may have contributed to persistent vertigo in some cases. Our sample included patients with particularly severe and refractory disease, reflected by higher average vertigo attack frequency compared to other series, which may limit generalizability to less severely affected populations.

Because of the retrospective design, certain potentially relevant data were unavailable, including standardized quality-of-life measures and information about concomitant or post-treatment Persistent Postural-Perceptual Dizziness (PPPD), which could contribute to persistent postoperative instability in some patients [38]. Finally, the therapeutic strategy adopted in our two centers may not be generalizable to all institutions. In both settings, endolymphatic sac surgery was favored over ablative options following failure of medical therapy and intratympanic corticosteroids, in accordance with a conservative, function-preserving approach. However, many centers routinely use chemical labyrinthectomy with intratympanic gentamicin as a competing second-line option, which may result in different surgical indications and patient profiles. These variations in clinical algorithms can affect cohort composition and limit broader applicability of our findings.

### 4.5. Strengths and Clinical Implications

Despite these limitations, our study has several strengths. The bicentric design with consistent treatment protocols enhances generalizability compared to single-center series. The detailed characterization of patients, including preoperative imaging, allows for comprehensive descriptive analysis. The inclusion of patients with non-serviceable hearing reflects real-world clinical practice where ELSS is considered for vertigo control even when hearing preservation is no longer the primary goal.

From a clinical perspective, our findings support several practical considerations. Endolymphatic sac was associated with vertigo control in approximately two-thirds of patients with refractory Ménière’s disease, with a favorable safety profile and low morbidity. The procedure preserves the potential for future interventions, an important consideration in stepwise management. Patients should be counseled about the significant risk of surgical ineffectiveness (approximately one-third in our series) and the lack of validated preoperative criteria for predicting outcomes. The progressive hearing decline observed over two years, whether due to surgery or natural disease progression, emphasizes the importance of long-term audiological monitoring after ELSS.

The exploratory association between longer disease duration and better outcomes, if validated in future studies, could have implications for timing of surgical intervention. However, this finding requires confirmation in larger prospective cohorts with prespecified analysis plans before influencing clinical decision-making. At present, surgical timing should be individualized based on symptom severity, quality of life impact, treatment failures, and patient preferences rather than disease duration alone.

## 5. Conclusions

Endolymphatic sac surgery effectively controlled vertigo in two-thirds of patients with medically refractory Ménière’s disease, with minimal morbidity. An exploratory post hoc analysis suggested a potential association between longer disease duration (>5 years) and improved outcomes, though this finding requires validation in independent cohorts before clinical application. Other preoperative factors investigated did not show significant associations with outcomes in this exploratory analysis. Progressive hearing decline was observed over two years, emphasizing the need for long-term audiological monitoring. Further prospective research with larger cohorts, prespecified analysis plans, and standardized patient-reported outcomes is needed to establish robust predictive criteria for patient selection and better characterize the contribution of natural disease history to observed surgical outcomes.

## Figures and Tables

**Table 1:** Population characteristics.

	Total	Success (A–B)	Failure (C–D–F)	
Sex				
Female	26/45 (57.8%)	19/30 (63.3%)	7/15 (46.7%)	
Male	19/45 (42.2%)	11/30 (36.7%)	8/15 (53.3%)	*p* = 0.29
Side				
Right ear	17/45 (37.8%)	13/30 (43.3%)	4/15 (26.7%)	
Left ear	28/45 (62.2%)	17/30 (56.7%)	11/15 (73.3%)	*p* = 0.28
Center				
Hospital 1	40/45 (88.9%)	26/30 (86.6%)	14/15 (93.3%)	
Hospital 2	5/45 (11.1%)	4/30 (13.3%)	1/15 (6.6%)	*p* = 0.51
Surgical technique				
EDB	34/45 (75%)	21/30 (70%)	13/15 (86.6%)	
ESS	10/45 (22.2%)	8/30 (26.6%)	2/15 (13.3%)	
ESD	1/45 (2.2%)	1/30 (3.3%)	0/15 (0%)	*p* = 0.43
Age (mean +/− SD)	54.4 +/− 12.6	54.3 (+/−12)	54.7 (+/−14.1)	*p* = 0.91
Mean duration of disease evolution before surgery (years +/− standard deviation)	7 (+/−7.6)	8.1 (+/−8.6)	4.5 (+/−4.4)	*p* = 0.14
Vestibular aqueduct width in preoperatively CT scan (mm)	2.9 (+/−2.0)	2.9 (+/−2.1)	2.8 (+/−2.0)	*p* = 0.83
Presence of migraine symptoms	11/45 (24%)	8/30 (27%)	3/15 (20%)	*p* = 0.72
Vertigo				
Mean number of vertigo attacks before surgery (+/−SD)	6.7 (+/−6.3)	7.1 (+/−6.7)	5.7 (+/−5.3)	*p* = 0.48
Presence of preoperative Tumarkin attacks	9/45 (20%)	7/30 (23.3%)	2/15 (13.3%)	*p* = 0.43
Hearing				
Mean pure tone average on preoperative audiometry (+/−SD)	59.7 (+/−17.5)	58.4 (+/−16.7)	62.3 (+/−19.3)	*p* = 0.49
Preoperative speech audiometry (%)	37.2 (+/−42.4)	39.5% (+/−42.5)	32.7 (+/−43.1)	*p* = 0.62
Presence of preoperative hearing fluctuations	29/45 (64.4%)	19/30 (63.3%)	10/15 (66.7%)	*p* = 0.83
Preoperative audiometry staging				
1	2/45 (4.4%)	2/30 (6.6%)	0/15 (0%)	
2	5/45 (11.1%)	4/30 (13.3%)	1/15 (6.66%)	
3	30/45 (66.7%)	19/30 (63.3%)	11/15 (73.3%)	
4	8/45 (17.8%)	5/30 (16.7%)	3/15 (20%)	*p* = 0.65
Total population size	45 (100%)	30/45 (66.7%)	15/45 (33.3%)	

**Table 2:** Success of surgery according to the duration of disease evolution before surgery.

	Success (A–B)	Failure (C–D–F)	Total	
<5 years	16/29 (55.2%)	13/29 (44.8%)	29/45 (64.4%)	
>5 years	14/16 (87.5%)	2/16 (13.3%)	16/45 (35.6%)	*p* = 0.029

**Table 3:** Success of surgery according to the pre-operative frequency of vertigo attacks.

	Success (A–B)	Failure (C–D–F)	Total	
<1 attack/week	13/30 (43.3%)	5/15 (33.3%)	18/45 (40%)	
1 to 2 attacks/week	8/30 (26.7%)	7/15 (46.7%)	15/45 (33.3%)	
>10 attacks/month	9/30 (30%)	3/15 (20%)	12/45 (26.7%)	*p* = 0.40

**Table 4:** Success of the surgery according to the curve shape of the preoperative audiometry.

	Success (A–B)	Failure (C–D–F)	Total	
Ascending curve	6/30 (20%)	3/14 (21.4%)	9/44 (20.4%)	
Descending curve	5/30 (16.6%)	2/14 (14.3%)	7/44 (15.9%)	
Flat curve	15/30 (50%)	5/14 (35.7%)	20/44 (45.4%)	
Bell-shape curve	4/30 (13.3%)	4/14 (28.6%)	8/44 (18.2%)	*p* = 0.45

**Table 5:** Success according to postoperative vertigo attacks or instability in the first weeks after surgery.

	Success (A–B)	Failure (C–D–F)	Total	
No vertigo attack	18/19 (94.7%)	1/19(5.2%)	19 (44.2%)	
Instability	5/9 (55.6%)	4/9 (44.4%)	9 (20.9%)	
Vertigo attacks	6/15 (40%)	9/15 (60%)	15 (34.9%)	*p =* 0.0023

**Table 6:** Pure tone and speech audiometry mean evolution across time periods.

	Postoperative	45 Days	6 Month	1 Year	2 Years
Bone-conduction Pure tone audiometry (dB)	−13.9 (+/−19.6)	−1.6 (+/−16.5)	−2.0 (+/−18.8)	−1.7 (+/−21.1)	−6.1 (+/−27.0)
Speech audiometry	NA	+2.1% (+/−48.8)	+2.6% (+/−46.0)	−10.2% (+/−49.8)	−18.6% (+/−43.2)

## Data Availability

https://doi.org/10.3886/E236962V2 (accessed on 7 January 2026).
